# A single amino acid change within the R2 domain of the VvMYB5b transcription factor modulates affinity for protein partners and target promoters selectivity

**DOI:** 10.1186/1471-2229-11-117

**Published:** 2011-08-23

**Authors:** Imène Hichri, Laurent Deluc, François Barrieu, Jochen Bogs, Ali Mahjoub, Farid Regad, Bernard Gallois, Thierry Granier, Claudine Trossat-Magnin, Eric Gomès, Virginie Lauvergeat

**Affiliations:** 1 Univ. de Bordeaux, Institut des Sciences de la Vigne et du Vin (ISVV), UMR 1287 Ecophysiologie et Génomique Fonctionnelle de la Vigne (EGFV), 210 Chemin de Leysotte, 33882 Villenave d'Ornon, France; 2 INRA, ISVV, UMR 1287 EGFV, 33882 Villenave d'Ornon, France; 3 ENITAB, ISVV, UMR 1287 EGFV, 33882 Villenave d'Ornon, France; 4 Department of Horticulture, Oregon State University, Corvallis, Oregon 97331, USA; 5 Dienstleistungszentrum Landlicher Raum (DLR) Rheinpfalz, Breitenweg 71, Viticulture and Enology group, D-67435 Neustadt/W, Germany; 6 Fachhochschule Bingen, Berlinstr. 109, 55411 Bingen am Rhein, Germany; 7 Université de Toulouse, INP-ENSAT Toulouse, Génomique et Biotechnologie des Fruits, Avenue de l'Agrobiopole BP 32607, 31326 Castanet-Tolosan, France; 8 Chimie et Biologie des Membranes et des Nanoobjets, UMR CNRS 5248, Bâtiment B14bis, Allée Geoffroy de Saint Hilaire, Université Bordeaux, 33600 Pessac, France

## Abstract

**Background:**

Flavonoid pathway is spatially and temporally controlled during plant development and the transcriptional regulation of the structural genes is mostly orchestrated by a ternary protein complex that involves three classes of transcription factors (R2-R3-MYB, bHLH and WDR). In grapevine (*Vitis vinifera *L.), several MYB transcription factors have been identified but the interactions with their putative bHLH partners to regulate specific branches of the flavonoid pathway are still poorly understood.

**Results:**

In this work, we describe the effects of a single amino acid substitution (R69L) located in the R2 domain of VvMYB5b and predicted to affect the formation of a salt bridge within the protein. The activity of the mutated protein (name VvMYB5b^L^, the native protein being referred as VvMYB5b^R^) was assessed in different *in vivo *systems: yeast, grape cell suspensions, and tobacco. In the first two systems, VvMYB5b^L ^exhibited a modified trans-activation capability. Moreover, using yeast two-hybrid assay, we demonstrated that modification of VvMYB5b transcriptional properties impaired its ability to correctly interact with VvMYC1, a grape bHLH protein. These results were further substantiated by overexpression of *VvMYB5b^R ^*and *VvMYB5b^L ^*genes in tobacco. Flowers from *35S::VvMYB5b^L ^*transgenic plants showed a distinct phenotype in comparison with *35S::VvMYB5b^R ^*and the control plants. Finally, significant differences in transcript abundance of flavonoid metabolism genes were observed along with variations in pigments accumulation.

**Conclusions:**

Taken together, our findings indicate that VvMYB5b^L ^is still able to bind DNA but the structural consequences linked to the mutation affect the capacity of the protein to activate the transcription of some flavonoid genes by modifying the interaction with its co-partner(s). In addition, this study underlines the importance of an internal salt bridge for protein conformation and thus for the establishment of protein-protein interactions between MYB and bHLH transcription factors. Mechanisms underlying these interactions are discussed and a model is proposed to explain the transcriptional activity of VvMYB5^L ^observed in the tobacco model.

## Background

MYB proteins represent a diverse and widely distributed class of eukaryotic transcription factors. In plants, *MYB *genes constitute a very large family encompassing 198 members in *Arabidopsis thaliana *for instance. Such large families are also observed in rice (*Oryza sativa *L.ssp. *indica*) and grape (*Vitis vinifera *L.), with no less than 85 and 108 members, respectively [1-3]. Plant MYB proteins are involved in the regulation of numerous physiological processes [4] and are for example notoriously known to regulate the phenylpropanoid pathway, allowing the biosynthesis of flavonoid, stilbenes and lignin compounds [4-7].

It is now well established that MYB proteins involved in the regulation of the anthocyanin and proanthocyanidin (PA) pathways act synergistically with bHLH partners (basic Helix Loop Helix) and WD-repeat proteins (WDR or WD40) to enhance the expression of structural genes (reviewed in [8-10]). Such tripartite MYB-bHLH-WDR (MBW) complexes were found to regulate anthocyanin biosynthesis in petunia flowers [11-13] and PA accumulation in *Arabidopsis *seed coat [14]. In grapevine, several branches of flavonoid biosynthesis are under the transcriptional control of different MYBs proteins [15-21]. Among them, two MYB transcription factors, VvMYB5a and VvMYB5b, contribute to the transcriptional regulation of the common parts of the pathway [20,21]. *VvMYB5b *is expressed in grape berry during PA synthesis in seeds and anthocyanin accumulation in skin. In tobacco, *VvMYB5b *ectopic expression resulted in accumulation of anthocyanins and PAs in flowers (stamens and petals), with no visible changes in vegetative organs [21]. As previously described in *Arabidopsis *and *Petunia*, MYB transcription factors require a bHLH partner for the trans-activation of flavonoid structural genes [17,21]. Recently, two bHLH transcription factors (VvMYC1 and VvMYCA1) and two WDR proteins (WDR1 and WDR2) have been identified in grapevine [22,23]. VvMYB5b interacts in yeast and *in planta *with VvMYC1 [22]. Thus, in grape berry, the interplay between each component of the MBW complex was proposed to control the spatiotemporal distribution of each class of flavonoid compounds. In this spatiotemporal control, three components must play a critical role: (i) the presence of the proteins at a given time in a given tissue, (ii) the DNA binding affinity of each of these proteins for their target genes, and (iii) the specific combination between partners that will result in the activation of a specific structural gene expression. Although the protein-protein interaction between MYB and bHLH proteins has been already investigated *in vitro *[24-26], the mechanisms underlying the formation of the whole MBW transcriptional complex have not been identified yet. In this complex, MYB proteins play a critical role in the determination of *cis*-elements and thus contribute to the selection of target genes. However, the affinity between MYB proteins and *cis*-elements may partly depend on the nature of the interacting bHLH partner, taking in account the fact that the interaction can modify the structural conformation of the MYB DNA-Binding domain [9,27-29].

MYB proteins are characterized by the presence of an extremely well conserved N-terminal domain that contains up to three imperfect R repeats (R1, R2 and R3) of about 53 amino acid residues each. These repeats, which contain three alpha-helices, adopt a common conformation named helix-turn-helix motives. Structural studies of three repeats in the vertebrate c-MYB have shown that both R2 and R3 are required for sequence-specific binding while R1 is not involved in the sequence recognition [30]. In each repeat, the three alpha-helices are stabilized by a hydrophobic core that includes three regularly spaced tryptophan residues. Within the R2 and R3 repeats, the C-terminal helix is involved in the DNA specific recognition process and the protein insertion into the DNA major groove. It has been suggested that the recognition helix of R3 specifically interacts with the core of the MYB-binding sequence (MBS). In contrast, the R2 C-terminal helix is supposed to interact less specifically with adjacent nucleotides [31-33]. Finally, the R3 repeat has also been proposed to provide a platform for protein-protein interactions, especially with bHLH cofactors [24].

Mutations altering protein-protein interactions between any member of the ternary complex without affecting their inherent properties (DNA binding activities and/or stabilization of the complex) not only will be of significant value in terms of improving fundamental knowledge of such protein complexes but may also be useful to propose innovative engineering strategies to enhance the biosynthesis of specific secondary metabolites in plant system models. In grapevine, the broader regulatory impact of VvMYB5b compared to more specific transcription factors such as VvMYBA1 or VvMYBPA1 and 2 makes it as potential candidate for such engineering strategy [21]. In this study, we investigated the consequences of a single amino-acid substitution located on the third helix of the R2 domain on the transcriptional regulatory properties of VvMYB5b [21]. Based on structural homology studies with the c-MYB protein, we choose to replace a positively charged arginine in position 69 from the native protein (VvMYB5b^R^) by a neutral leucine (VvMYB5b^L^). Effects of conformational changes on the DNA-binding and the *trans*-regulation properties of the mutated VvMYB5b^L ^protein were investigated in yeast and in grape suspension cells and compared to those of the native protein. VvMYB5b^R ^and VvMYB5b^L ^capabilities to physically interact with the bHLH protein VvMYC1 were assessed using two-hybrid assays in yeast. Finally, overexpression of VvMYB5b^L ^in tobacco was performed to estimate the *in planta *impact of the mutation on the array of VvMYB5b^R ^target genes. Taken together, our results highlight the importance of dimerization between MYB and bHLH factors for the selectivity of target genes.

## Results

### Structural model of VvMYB5b R2R3 domain

**Figure 1:**
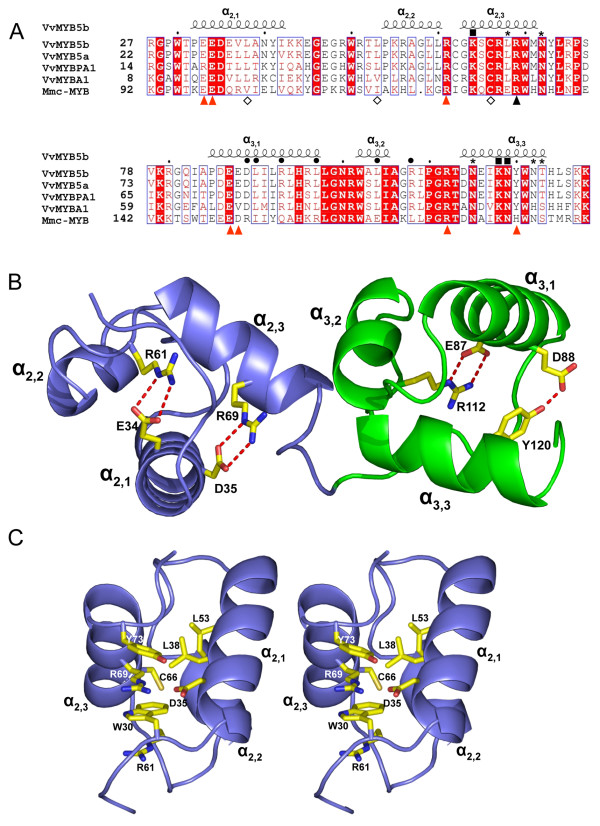
**Structure of the R2R3 domain of different MYB proteins**. (A) Protein sequence alignment of the R2R3 domain of grapevine MYB transcription factors regulating the flavonoid pathway and mouse (*Mus musculus*) c-MYB. GenBank accession numbers are indicated below: VvMYB5b (AY899404), VvMYB5a (AY555190), VvMYBPA1 (AM259485), VvMYBA1 (AB097923), and Mmc-MYB (NP_034978). Identical residues are shown in white on a red background, and conserved residues are red. The R/L mutation is indicated with a dark triangle, residues interacting with DNA bases [30,35] are indicated with either a dark square or an asterisk for strong and weak interactions respectively. Dark circles denote residues interacting with bHLH partners [24]. Diamonds denote residues involved in the hydrophobic pocket in domain R2 and amino acids involved in salt bridge interactions in Mmc-MYB [30] are highlighted with red arrow heads. This figure was drawn using web ESPript [61]. (B) R2 and R3 domains of the VvMyb5b modeled structure obtained deduced from the X-ray diffraction structure of the mouse c-MYB proto-oncogene R2-R3 domain (pdb entry code 1gv2). The figure was drawn with PyMOL [62]. (C) Stereo view of the environment of residue R69 within the R2 domain.

The *Vitis vinifera MYB5b *gene encodes a MYB-like protein containing two imperfect repeats (R2R3) and an interaction domain ([D/E]Lx_2_[R/K]x_3_Lx_6_Lx_3_R) with bHLH protein partners [21,24,34] (Figure 1A). The alignment of the VvMYB5b sequence with MYB transcription factors already characterized in grape (VvMYB5a, VvMYBA1, and VvMYBPA1) confirms the high sequence homology of the MYB domains (Figure 1A). The sequence identity remains very high (46%) when compared with the R2 and R3 repeats of mouse c-MYB, a protein with its 3D structure already characterized in its free state or in complex with DNA [30]. Groups of highly conserved residues have been assigned key roles in the structure and function of these proteins: a first group of residues located at the C-terminal parts of the R2 and R3 domains is involved in interactions with DNA. A second group, located at the N-terminal part of domain R3, interacts with bHLH protein partners as described above [24,30,35]. Finally, a third group includes residues responsible for the ternary structure of the protein: in each domain, several charged residues establish salt bridges between α-helices which maintain their relative orientations, whereas hydrophobic residues form a hydrophobic core buried within the three α-helices [36].

A structural model of VvMYB5b was built (Figure 1B) using the crystallographic coordinates of the Mmc-MYB R2-R3 domain (pdb code: 1gv2) as starting model. The resulting model appears very close to the template model with a root-mean-square deviation (rmsd) of superimposed Cα of 0.89 Å for 100 aligned residues. As visualized in Figure 1B, all four salt bridges observed in Mmc-MYB are strictly conserved in VvMYB5b and adopt the same conformations, with the exception, in domain R3, of the interaction D88-Y120, which is substituted by a D152-H184 interaction in the Mmc-MYB protein. Within domain R2, residue R69 is involved in a conserved salt bridge and was chosen as a target for single point mutation for the following reasons: (i) the salt bridge appears to be strictly conserved in all MYB sequences (Figure 1A) and does not interact with bHLH partners [24]; (ii) its counterpart in Mmc-MYB (R133) was shown to interact with phosphate groups of target DNA [30] to facilitate DNA binding; (iii) D35, the partner of R69 in the salt bridge, appears to be far enough from any other residue from the R2 domain C-terminal α-helix to avoid establishing a new stabilizing interaction. In addition, R69 also takes part in the stacking of several side chains, i.e. R61, W30, R69 and Y73, which certainly participates to the 3D structure arrangement of the R2 domain (Figure 1C). A similar situation has been observed in Mmc-MYB with the residues R125, W95, R133 and H137.

Therefore, the arginine in position 69 of VvMYB5b was replaced by a leucine neutral residue. The resulting mutation, named R69L and located nearby the DNA Binding Domain (DBD), appeared likely to modify the interaction with the DNA backbone and the protein activity by disrupting the ternary structure of the transcription factor itself.

### The R69L mutation reduces VvMYB5b trans-activation capacity in yeast

**Figure 2:**
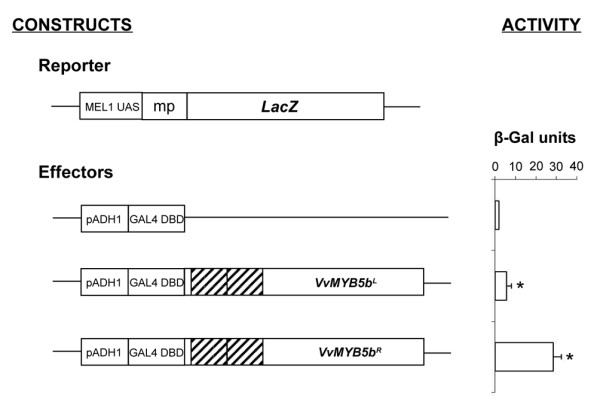
**The single residue substitution R69L reduces VvMYB5b trans-activation capacity in yeast**. VvMYB5b^R ^and VvMYB5b^L ^coding sequences were fused to GAL4 DNA Binding Domain (DBD) and their ability to activate *LacZ *reporter gene expression was quantified using β-galactosidase activity measurements. Each value is the mean ±SD of two independent yeast transformations and each experiment included three measures (Student's *t *test; * *P *< 0,05 vs. negative control). Constructs are identified as indicated to the left of the figure. MEL1 UAS, Melibiose 1-GAL4 Upstream Activating Sequence; mp, minimal promoter; pADH1, Alcohol Dehydrogenase 1 promoter. Both MYB repetitions (i.e. R2 and R3 repeats) are indicated using dashed boxes.

An assay was conducted to determine whether the R69L mutation affects VvMYB5b trans-activation properties in yeast. As shown in Figure 2, yeasts transformed with the *VvMYB5b^R ^*effector construct exhibited a 5-fold increase in β-galactosidase activity compared to yeasts that express VvMYB5b^L^. Nevertheless, VvMYB5b^L ^was still functional despite a growth delay on solid selective medium (6 days) compared to VvMYB5b^R ^recombinant yeasts that were able to develop 4 days after transformation (data not shown). Indeed, VvMYB5b^L ^could activate *LacZ *expression 3 times more than the GAL4-DBD itself. These results indicate that (i) VvMYB5b can activate transcription in yeast and (ii) that the R69L substitution significantly reduces VvMYB5b transcriptional activities.

### VvMYB5b^L ^no longer activates transcription of a flavonoid structural gene in grape cells

As for many other MYB proteins, VvMYB5b requires co-expression of both bHLH and WDR protein partners, i.e. AtEGL3 (ENHANCER of GLABRA 3) and AtTTG1 respectively, to up-regulate target gene expression [15,17,21,34]. Thus, a dual luciferase assay was conducted to assess the effect of the R69L substitution on VvMYB5b ability to activate the *VvCHI *promoter in grape cells, in the presence or the absence of bHLH and WDR proteins.

**Figure 3:**
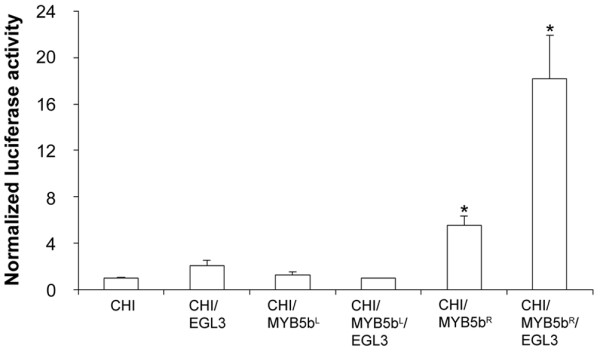
**Unlike VvMYB5b^R^, VvMYB5b^L ^is not able to activate *VvCHI *promoter in grape cells**. Results of transient expression after co-bombardments of cultured grape cells with the *Firefly *luciferase reporter gene fused to the *VvCHI *promoter and combinations of VvMYB5b^R ^or VvMYB5b^L^, together with AtEGL3 and AtTTG1. The normalized luciferase activity was calculated as the ratio between the *Firefly *and the *Renilla *luciferase (used as internal control) activity [63]. All bombardments included the WD40 protein AtTTG1 (GenBank accession number AJ133743). Values indicate the fold increase relative to the activity of the *VvCHI *promoter transfected without transcription factors. Each column represents the mean value ±SD of three independent experiments (Student's *t *test; * *P *< 0.05 vs. *VvCHI *alone).

As shown in Figure 3, co-transformation with VvMYB5b^R ^effector plasmid and *VvCHI *reporter construct, together with the WD40 protein AtTTG1, resulted in a 5-fold increase of luciferase activity, as compared to the control (reporter construct with AtTTG1). Presence of AtEGL3 increased the transcriptional activity of VvMYB5b^R ^up to 18-fold. In contrast, same experiments with VvMYB5b^L ^showed that VvMYB5b^L ^was not able to activate *VvCHI *promoter in the presence of AtTTG1 (Figure 3). In the same way, co-transformation using *VvMYB5b^L ^*construct with AtEGL3 and AtTTG1 did not increase the luciferase activity. Altogether, these results show that, in grapevine cells, VvMYB5b^L ^no longer displayed any transcriptional activation of the *VvCHI *promoter in the presence of the two imposed proteins from Arabidopsis, AtEGL3 and AtTTG1.

### The R69L substitution abolishes VvMYB5b interaction with a bHLH partner

**Figure 4:**
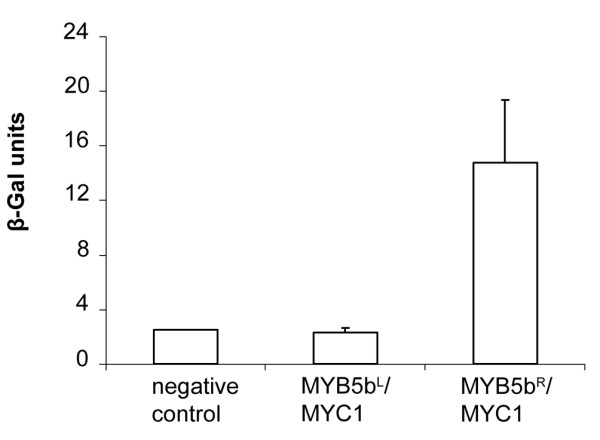
**VvMYB5b^L ^loses its ability to physically interact with the bHLH transcription factor VvMYC1 in yeast**. Yeast two-hybrid experiments have been performed by co-transformation with VvMYB5b^R ^or VvMYB5b^L ^proteins fused to GAL4 Activation Domain, and VvMYC1 fused to GAL4 DNA Binding Domain. Transformed yeasts were selected on SD-Leu^-^Trp^- ^medium and tested for *LacZ *activation. β-Galactosidase activity results are the mean of three measurements of three independent yeast clones. Negative two-hybrid control refers to the control provided by the manufacturer. Error bars indicate SD.

A yeast two-hybrid assay was conducted to investigate the ability of VvMYB5b^L ^to physically interact with a putative *Vitis *bHLH partner. Our results (Figure 4) confirmed that VvMYB5b^R ^could interact with VvMYC1, as previously described [22]. On the other hand, VvMYB5b^L ^was not able to form dimers with VvMYC1 to activate *LacZ *expression.

In addition, the ability of the VvMYB5b^R ^and VvMYB5b^L ^proteins to bind MBS (MYB binding sites) *cis*-elements was evaluated using EMSA (Electrophoretic Mobility Shift Assay). Both proteins were synthesized by an *in vitro *transcription and translation assay and biotinylated protein bands were detected by a chemiluminescent assay (see additional file 1). The results showed that both proteins accumulated in identical ways and are not degraded. However, neither native VvMYB5b^R ^nor mutated VvMYB5b^L ^could bind MBS sequences using EMSA. Likewise, none of both proteins (VvMYB5b^L^, VvMYB5b^R^) was able to bind the *VvCHI *promoter sequence in yeast one-hybrid experiments (data not shown).

### Flavonoid biosynthesis genes are differentially expressed in flowers of *VvMYB5b^R ^*or *VvMYB5b^L ^*transgenic tobacco lines

**Figure 5:**
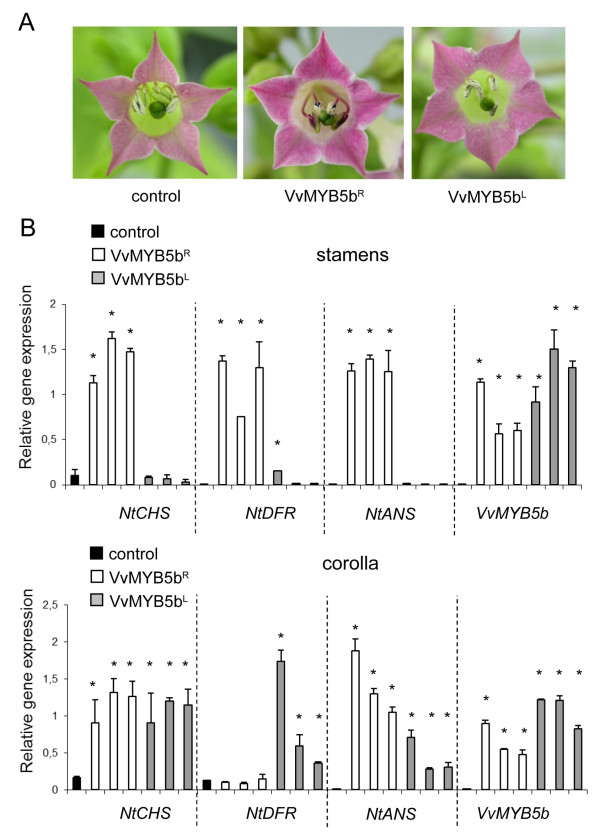
**Analysis of VvMYB5b^R ^and VvMYB5b^L ^ectopic expression effect in tobacco plants flowers**. (A) Flowers of *VvMYB5b^R ^*overexpressing plants showed an intense red coloration of petals and stamens, compared to control and *VvMYB5b^L ^*transgenic flowers. (B) Real time quantitative RT-PCR analysis of *NtCHS *(chalcone synthase), *NtDFR *(dihydroflavonol reductase) and *NtANS *(anthocyanidin synthase) transcript abundance in stamens and corollas. Gene expression is shown relative to *NtUbiquitin *transcript levels in each sample. Results are presented for three independent transgenic lines overexpressing either *VvMYB5b^R ^*or *VvMYB5b^L^*, and compared to control plants. *VvMYB5b *indicate transgene transcript levels. Each bar represents the mean ±SD of three replicates (* *P *< 0.05 vs. control plants according to the ANOVA).

*VvMYB5b^R ^*and *VvMYB5b^L ^*coding sequences were ectopically expressed in tobacco plants under the control of the *35S *constitutive promoter. Three T2 homozygous independent lines tested for each construct were used for further investigations. Analyses were only carried out on flowers since no phenotypic differences were detected at the vegetative level. Corolla and stamens of *35S::VvMYB5b^R ^*tobacco flowers exhibited a strong red pigmentation and a purple color, which was associated with higher anthocyanidin accumulation not observed in control plants [21]. By contrast, flowers of tobacco plants over-expressing *VvMYB5b^L ^*did not exhibit a greater accumulation in anthocyanidin in both flower organs (Figure 5A) and no significant changes of anthocyanin content were observed in corolla and stamens (see additional file 2). To tentatively explain these phenotypes, transcript abundances of three tobacco flavonoid biosynthetic genes (*chalcone synthase *(*NtCHS*), *dihydroflavonol 4-reductase *(*NtDFR*) and *anthocyanidin synthase *(*NtANS*)) were monitored by quantitative RT-PCR (qRT-PCR) to identify *in planta *target structural genes of VvMYB5b together with the impact of the mutation on the expression of these same genes. As shown in Figure 5B, none of these genes was expressed in stamens of control plants, which is consistent with the fact that anthocyanins are not normally synthesized in this particular tissue. As previously described in [21], overexpression of *VvMYB5b^R ^*induced higher transcription of *NtCHS, NtDFR *and *NtANS *mRNAs together with anthocyanin accumulation in stamens. In corolla cells, *NtDFR *expression did not appear to be affected but an increase in *CHS *and *ANS *transcript abundances was observed and correlates with an anthocyanin content significantly higher than in control plants.

In contrast, *VvMYB5b^L ^*overexpression did not enhance *NtCHS*, *NtANS *and *NtDFR *transcript abundances in stamens although expression levels of transgene for both constructs (*35S::VvMYB5b^R ^*and *35S::VvMYB5b^L^*) were the same. However, VvMYB5b^L ^appeared to retain some *trans*-activation activity in corolla where *NtCHS *and *NtANS *transcripts abundance was significantly higher than in wild-type plants. In addition, corolla cells expressing VvMYB5b^L ^accumulated significantly more *NtDFR *transcripts than control and *35S::VvMYB5b^R ^*plants. Surprisingly, this increase in flavonoid genes expression did not affect the anthocyanin concentration in *VvMYB5b^L ^*corolla (see additional file 2). Altogether, these results indicate that (i) VvMYB5b^L ^has severely lost its trans-activation ability in stamens whereas this same regulatory protein was still active in corolla; (ii) VvMYB5b^L ^might have new regulatory functions in corolla cells as its overexpression induced the up-regulation of the *NtDFR *that was not observed in *35S::VvMYB5b^R ^*plants.

## Discussion

Over the past two decades, an increasing number of studies investigating the transcriptional regulation of the flavonoid pathway have been published (reviewed in [8,10]). Most of them emphasized the pivotal role of MYB transcription factors in the control of this metabolic pathway. More recently, new findings highlighted the importance of a multi-protein complex involving MYB proteins with bHLH and WDR partners in the coordination of the transcriptional regulation of flavonoid biosynthetic genes. Nevertheless, the way in which this multi-protein complex specifically regulates expression of genes depending on the tissue, the developmental stage or the environmental conditions is not fully understood yet.

The structure of the MYB DNA-Binding Domain (DBD) interacting with a double DNA strand has already been investigated in several models [37-39]. These studies have shown that the third helices of both R2 and R3 are involved in the recognition of a specific DNA consensus sequence [30,40]. In Mmc-MYB, K128, positioned in the R2 domain, together with K182 and N183 positioned in the R3 domain, were identified as key residues in the 'recognition' of the specific nucleotide sequence AACNG, the so-called 'MYB Binding Site' [30,41]. Later, the same authors demonstrated that the methylene chain of residue R133 delimits, with three other amino acids (V103, C130 and I118), a cavity in the centre of a hydrophobic core that may play a role in the conformational stability of the R2 domain [36]. For instance, an amino acid substitution (V103L) within this cavity reduces the conformational flexibility of the R2 domain and thereby significantly decreases specific MYB-DNA binding activity and trans-activation. The model of the VvMYB5b R2R3 domain illustrated in Figure 1B shows that the R69 residue is, like its counterpart R133 in Mmc-MYB, involved in the formation of a salt bridge that may participate in the stabilization of the protein [30]. The impact of salt bridges formation in the activity of such transcription factor is poorly understood, but the few available studies suggest that they may influence both DNA binding affinities and trans-activation properties of transcription factors. Disruption of the salt bridge by amino acid substitution affected the CRP (cAMP Receptor Protein) protein activity and led to a reduction of the *Lac *promoter trans-activation, without affecting its DNA binding affinity [42,43]. This reduction is attributed to an alteration of the interaction with the α-subunit of RNA polymerase. In our study, R69 was substituted by a leucine residue, and we demonstrated that this single residue mutation in the third helix of the R2 repeat could modify the protein interaction properties of VvMYB5b together with its DNA binding affinities.

### The R69L substitution affects trans-activation properties of VvMYB5b

In yeast, we found that VvMYB5b^L ^effector construct fused to yeast GAL4-DBD was barely able to increase the expression of reporter genes. One can make the assumption that the amino acid substitution within R2 repeat in VvMYB5^L ^may result in a weaker interaction between this protein and yeast general co-activators of the RNA polII complex. Indeed, transcription factors act in several ways through protein interactions to enhance the expression of a target gene. Activators interact with chromatin remodelling factors, general transcription factors (GTFs) of the RNA polII pre-initiation complex, and can also affect initiation of the transcription and elongation [44,45]. The decrease of transactivation properties of VvMYB5b caused by the mutation, in yeast, can be explained by a decrease of its ability to recruit the yeast GTFs.

In eukaryotic transcription factors, DNA-Binding Domains and Activation/Repression domains are thought to be spatially independent. The yeast two-hybrid technique is based on this concept [46]. Based on our results (Figure 2), these two domains seem to be intimately dependent, as previously shown for some MYB transcription factors. In the c-MYB protein for instance, the C-terminal negative regulation domain can interact with the R2R3 N-terminal domain to alter its intrinsic properties [47]. Likewise, in C1, a MYB transcription factor promoting anthocyanin accumulation in maize, the R2R3 domain seems to interact with the C-terminal region to keep the protein inactive in the absence of its bHLH partner [25].

Although VvMYB5b works in yeast as a strong transcriptional activator, it requires in grape cells, as does VvMYBA, at least one bHLH partner to be fully functional [15, 21, 22, present work]. In this study, VvMYB5b^L ^was not able to activate *VvCHI *promoter in grape cells despite the co-expression of both bHLH and WDR. In addition, we show that, unlike VvMYB5b^R^, VvMYB5b^L ^did not interact with VvMYC1 in yeast [22]. Taken together, these results suggest that the amino acid substitution clearly has an impact on the protein-protein interaction selectivity and subsequently on the trans-activation properties of the regulatory complex as well.

### The R69L mutation modifies the *in vivo *selectivity of VvMYB5b for protein partners

Overexpression experiments in tobacco suggest the presence of different regulatory mechanisms in stamens and corollas, with regard to flavonoid pathway genes expression. First, none or little expression was observed for the *NtCHS*, *NtANS *and *NtDFR *genes in stamens of control plants. This suggests the absence of an efficient regulatory complex in this tissue or the lack of at least one component of the system. However, in corollas of control plants, a baseline expression was detected for the same structural genes on the same control plants supporting the idea of a pre-existing transcriptional network regulating the accumulation of anthocyanins in these floral organs.

**Figure 6:**
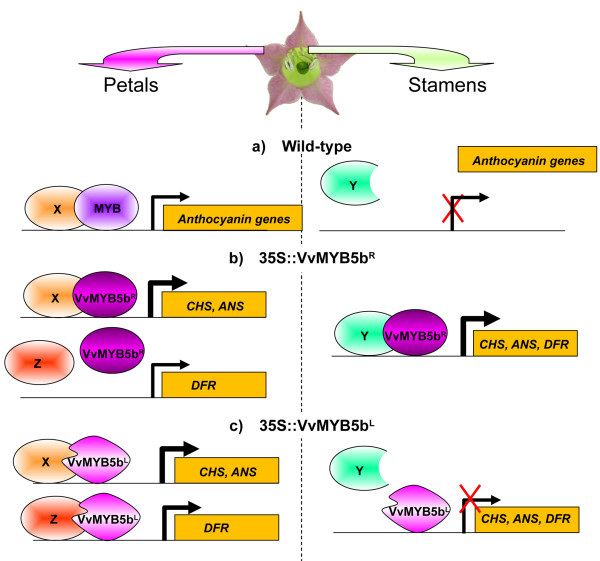
**Proposed model for effect of the R69L substitution on interaction specificity with protein partners and consequently on trans-activation properties of VvMYB5b in tobacco flowers**. X, Y and Z indicate endogenous transcription factors expressed in corolla and/or stamens of tobacco flowers. MYB is a tobacco endogenous transcription factor normally expressed in petals and involved in anthocyanin synthesis in cooperation with endogenous partner, such as a bHLH protein. In transgenic petals, both VvMYB5b (mutated or normal) are able to recognize endogenous partners and to activate promoters of CHS and ANS encoding genes. In the particular case of *NtDFR *promoter, our results suggest the R69L mutation may change the DNA binding specificity of the protein complex, because VvMYB5b^L ^activated *NtDFR *transcription, contrary to VvMYB5^R^. In wild-type stamens, anthocyanin biosynthetic pathway is not active, but transcription factors (Y) involved in other processes should be present. In transgenic stamens, VvMYB5b^R ^may be able to recognize this(ese) partner(s) to activate promoters, while VvMYB5b^L ^may not. Putative WDR factors, which have been shown in numerous models to be part of the complex, are not indicated in the figure.

In *35S::VvMYB5b^R ^*transgenic tobacco stamens, it appears that the presence of the native VvMYB5b^R ^protein and its interaction with endogenous pre-existing protein partner(s) leads to the activation of the entire anthocyanin biosynthetic pathway ([21]; Figure 6). In corollas, the absence of *NtDFR *upregulation observed in *35S::VvMYB5b^R ^*plants might be explained by the lack of interaction between VvMYB5b^R ^and a specific protein partner different from the one required for *NtANS *and *NtCHS *genes expression (termed Z in Figure 6). Another hypothesis may involve the presence of two distinct *NtDFR *genes in stamen and corolla, respectively. This alternative explanation cannot be totally ruled out but seems unlikely, taking into account the fact that the primers used in this study have been designed to amplify the two *DFR *genes identified to date in the tobacco genome.

In the *35S::VvMYB5b^L ^*plants, the clearly different behavior of VvMYB5b^L ^in stamen and corolla cells regarding gene activation capabilities supports the hypothesis of the presence of various protein partners in these tissues. In addition, the induction of *NtCHS*, *NtANS *and *NtDFR *genes expression observed in corolla indicates that VvMYB5b^L ^can efficiently bind DNA in this tissue. Thus, in stamens, VvMYB5b^L ^might fail to interact with the endogenous co-partner(s), and thus not induce the expression of the *NtCHS*, *NtANS *and *NtDFR *genes (Figure 6). The situation is clearly different in corollas where the presence of VvMYB5b^L ^leads to the induction of all genes studied, indicating that the mutated protein can interact with the array of endogenous co-partners needed for the activation of *NtCHS*, *NtANS *and *NtDFR *genes expression. In addition, the induction of *NtDFR *expression in corolla cells, which is not observed in the presence of VvMYB5b^R^, indicates that the structural changes linked to the mutation have now allowed the interaction with the specific partner required for *NtDFR *gene expression (Figure 6). Thus, taken together, these results indicate that the R69L substitution modifies the interaction capabilities of VvMYB5b with its putative protein partners, which subsequently impacts on the regulation of target genes expression.

In maize, amino acid substitutions within the DNA binding domain of the MYB transcription factor ZmP1 also has a strong influence on the cooperative effect of ZmP1 with its partners [25]. Indeed, ZmP1 does not require the interaction with the bHLH protein R to transactivate the *DFR *gene but fails to transactivate the *bz1 *gene encoding UDP-glucose:flavonoid 3-*O*-glucosyltransferase [24,25]. Mutation of ZmP1 within the DBD facilitates ZmP1 interaction with R, which in turn allows the binding of the complex to the promoter region of *bz1 *gene.

Further investigations will be needed to ascertain the model presented in Figure 6, such as the identification of different bHLH or WDR partners in both tobacco corollas and stamens. Co-expression of two different *bHLH *genes has already been demonstrated in petunia flowers, where *AN1 *and *Jaf13 *are preferentially expressed in corolla and stamens, respectively [11,48]). Likewise, in snapdragon flowers, the MYB transcription factors Rosea1, Rosea2 and Venosa control anthocyanin biosynthesis by differentially interacting with the bHLH partners Mut and Delila in the different floral organs [49]. In the same way, the *Gerbera hybrida *bHLH protein GMYC1 is thought to control the expression of the *GhDFR *gene in corolla and carpel tissues, whereas an alternate GMYC1-independent regulatory mechanism may exist in pappus and stamens [50]. These studies indicate that different bHLH transcription factors may be co-expressed in the different tissues of tobacco flowers. However, for this plant species, only one MYB transcription regulating the flavonoid pathway factor has been characterized so far [51].

## Conclusions

The amino acid substitution in position 69 was expected to have an impact on the DNA-binding activity of VvMYB5b^L^, as previously described for the c-MYB protein [30,52]. According to our results, neither native VvMYB5b^R ^nor mutated VvMYB5b^L ^were able to bind MBS sequences in EMSA experiments. However, VvMYB5b^R ^did activate the *VvCHI *promoter when co-expressed with the co-factors AtEGL3 (bHLH) and AtTTG1 (WDR) in grapevine cells (Figure 3), but was not able to bind the same sequence in yeast one-hybrid experiments. These results indicate that VvMYB5b needs its protein partner(s) to bind DNA and that EMSA and yeast one-hybrid methods are not appropriate to investigate the ability of VvMYB5b^R/L ^to bind target sequences. Finally, the upregulation of the *NtCHS*, *NtANS *and *NtDFR *genes observed in 35S::*VvMYB5b^L ^*tobacco plants is consistent with the presence of a functional VvMYB5b^L ^protein. Thus, VvMYB5b^L ^appears still able to recognize and bind DNA, even though further investigations will be needed to ascertain the direct or indirect role of residue R69 in the DNA binding properties of VvMYB5b.

In summary, this work describes the structural and biological consequences of a single amino acid change on both the dimerization and the DNA binding properties of a grapevine MYB transcription factor. These two functions appear related, as the conformation of the R2R3 domain, that regulates DNA affinity and binding, can be modified after interactions with protein partners. As a consequence, the array of target genes of a given MYB factor may vary depending on the protein partner involved.

## Methods

### Plant Material

Seeds from wild type and homozygous T2 generation of transgenic tobacco plants (*Nicotiana tabacum *cv Xanthi) were sterilized in 2.5% potassium hypochlorite, 0.02% Triton X-100 for 10 min, and washed five times with sterile water. After cold treatment at 4°C for 48 h, seeds were germinated on MS medium [53] containing 3% (w/v) sucrose, supplemented with 200 μg/ml kanamycin for transgenic plants, at 25/20°C under a 16 h light/8 h dark regime. Eight weeks after germination, *in vitro *grown plantlets were transferred to soil into individual pots and cultivated in a growth chamber under the same environmental conditions. The suspension culture of grapevine Chardonnay (*Vitis vinifera *L.) petiole callus was grown in grape Cormier medium as described in [54], at 25°C in darkness on an orbital shaker at 90 rpm.

### VvMYB5b R2R3 domain modeling

*VvMYB5b *was modeled starting from the crystal structure of the mouse c-MYB R2R3 domain (PDB code 1GV2, Tahirov et al., unpublished result) using the SWISS-MODEL server [55]. The obtained model was further checked using the molecular graphics program COOT [56]. Misorientation of a few side chains has been manually corrected and the full model regularized by molecular dynamics simulated annealing, using the standard protocols implemented with the Phenix software [57].

### Generation of the VvMYB5b^L ^substitution and tobacco stable transformation

The *VvMYB5b *cDNA sequence (gene accession AY899404) used in this study was previously inserted in the pGEM-T-Easy cloning vector (Promega, Madison, WI) [21]. The R69L substitution was introduced into the cloned *VvMYB5b *using the QuickChange site-directed mutagenesis kit (Stratagene). Reactions were carried out using the following primer pair: 5'-CAAGAGCTGTCGCCTCCTCTGGATGAACTACCTC-3' (sense) and 5'-GAGGTAGTTCATCCAGAGGAGGCGACAGCTCTTG-3' (antisense). The presence of the introduced mutation in the cDNA was confirmed by DNA sequencing. The native *VvMyb5b^R ^*and *VvMYB5b^L ^*full length cDNAs were then cloned between the *Xba*I/*Sac*I restriction sites of the pGiBin19 binary vector between the *35S *promoter of the cauliflower mosaic virus and the *nopaline synthase *(*nos*) poly(A) addition site, as described in [21]. Both constructions were introduced into *Agrobacterium tumefaciens *LB4404 host strain. Tobacco was transformed and regenerated according to the leaf discs method [58]. Selection of the primary transformants was carried out on MS medium containing 200 μg/ml kanamycin. Presence of the transgene was confirmed by PCR on genomic DNA extracted from leaves of primary transformants, according to the manufacturer instructions (DNeasy Plant Mini Kit, Qiagen). Seeds of self-fertilized T1 and T2 lines were collected and single-copy insertion T2 lines were selected based on a Mendelian segregation ratio.

### RNA extraction and gene expression analysis

Total RNA was isolated from wild-type and transgenic tobacco flower tissues according to [59]. At least three flowers were randomly collected per plant, and two plants selected for each lines: control (untransformed plants), *35S::VvMYB5b^R ^*and *35S::VvMYB5b^L^*. One μg of total RNAs was reverse transcribed with oligo(dT)12-18 in a 20 μl reaction mixture using the Moloney murine leukemia virus (M-MuLV) reverse transcriptase (RT) according to the manufacturer's instructions (Promega, Madison, WI). Transcript levels of *NtCHS*, *NtF3H *and *NtDFR *endogenous genes and the transgene *VvMYB5b^R/L ^*were measured by real-time quantitative RT-PCR, using SYBR Green on an *iCycler iQ^® ^*(Bio-Rad) according to the procedure described by the supplier. PCR reactions were performed in triplicate using 0.2 μM of each primer, 5 μl SYBR Green mix (Bio-Rad) and 0.8 μl DNAse treated cDNA in a final volume of 10 μl. Negative controls were included in each run. PCR conditions were: initial denaturation at 95°C for 90 s followed by 40 cycles of 95°C for 30 s, 60°C for 1 min. Amplification was followed by melting curve analysis to check the specificity of each reaction. Data were normalized according to the *NtUbiquitin *gene expression levels and calculated with a method derived from the algorithms outlined by [60]. Statistical analysis of the data was performed by analysis of variance (ANOVA) test using Sigma-Plot software. Sequences of the primers used for quantitative RT-PCR are indicated in Table 1. Two highly homologous sequences encoding DFR were used to design primers for real-time quantitative RT-PCR experiments in tobacco (accession numbers: EF421430 and EF421429).

**Table 1:** Primers used for real-time quantitative RT-PCR analysis.

Gene	Accession	Sequence (5'-3')	Amplified fragments size (bp)
*VvMYB5b*	AY899404	F: GCCATGACTTCCACGTCTG R: CATTGCAGGGTGTTGAAGCC	115

*NtCHS*	AF311783	F: GGTTTGGGAACTACTGGTG R: CCCACAATATAAGCCCAAGC	126

*NtANS*	EB427369	F: TCCATCTGGCCTAAAATCCCT R: AACGCCAAGTGCTAGTTCTGG	226

*NtDFR*	EF421429	F: CGCGTCCCATCATGCTATC R: AATACACCACGGACAAGTCC	116

*NtUbiquitin*	NTU66264	F: GAAAGAGTCAACCCGTCACC R: GAGACCTCAAGTAGACAAAGC	138

### Co-transfection experiments and dual luciferase assays

*VvMyb5b^R ^*and *VvMyb5b^L ^*full length cDNAs were amplified by PCR using the Phusion™ High-Fidelity DNA polymerase (Finnzymes) with oligonucleotides introducing the *Bam*HI (5'-TAAT*GGATCC*ATGAGGAATGCATCCTCA-3') and *Sal*I (5'-TAAT*GTCGAC*TCAGAACCGCTTATCAGGTTG-3') restriction sites (indicated in italics), and cloned in the pDH5 vector (kindly given by Pr M. Hernould, Bordeaux, France), a derivative from the pUC18 vector, that allows constitutive transient expression of the transgene. Integrity of each coding sequence was verified by sequencing (MWG, France) using the pDH5F (5'-CCCACTATCCTTCGCAAG-3') and the pDH5R (5'-CTAATTCCCTTATCTGGGAA-3') primers. Transient co-transfection experiments of grapevine suspension cells and dual-luciferase assays were carried out as previously described in [17].

### Beta-galactosidase assay in yeast

*VvMyb5b^R ^*and *VvMyb5b^L ^*cDNAs were amplified by PCR using the Phusion™ High-Fidelity DNA polymerase (Finnzymes) using oligonucleotides introducing the *BamH*I restriction site (indicated in italics) at the 5' (5'-TAAT*GGATCC*AGATGAGGAATGCATCCTCA-3') and 3' (5'-TAAT*GGATC*TCAGAACCGCTTATCAGGTTG-3') ends. After enzymatic digestion, PCR fragments were introduced into the pGBKT7 vector (Clontech, BD Bioscience), in fusion with the GAL4 DNA Binding Domain (DBD) coding region, under the control of the *ADH1 *promoter. pGBKT7 vector carries the Kan^R ^for selection in *E. coli *and the *TRP1 *nutritional marker for yeast selection. The yeast strain AH109 was independently transformed with pGBKT7, pGBKT7-*VvMYB5b^R^*, or pGBKT7-*VvMYB5b^L ^*using the PEG/LiAc method, based on the manufacturer's instructions. Transformants were selected on synthetic dropout (SD) media lacking tryptophan (SD-Trp^-^) or histidine, adenine, and tryptophan (SD-His^-^Ade^-^Trp^-^). In parallel, positive and negative controls of interaction, provided by the manufacturer, have been performed (BD Matchmaker™ Library Construction & Screening Kit, Clontech, BD Bioscience). As *LacZ *constitutes the fourth reporter gene of this system, β-galatosidase activity was monitored in recombinant yeasts grown on selective medium. β-galactosidase assays with the ONPG (O-nitrophenyl-β-D-galactopyranoside) substrate were performed following the manufacturer instructions. Relative β-galactosidase activity was obtained after normalization with the optical density at 600 nm.

### Yeast two-hybrid assay

*VvMYB5b^R ^*or *VvMYB5b^L ^*coding regions were fused to the GAL4 Activation Domain (AD), and the coding sequence of *VvMYC1 *was fused to the GAL4-DBD. Because of its intrinsic ability to activate transcription in yeast, VvMYB5b^R ^has not been fused to GAL4-DBD, and consequently the reciprocal combinations were excluded from the two-hybrid assay. *VvMyb5b^R ^*and *VvMyb5b^L ^*cDNAs were amplified by PCR using the Phusion™ High-Fidelity DNA polymerase with specific primers containing anchors: F, 5'-TTCCACCCAAGCAGTGGTATCAACGCAGAGTGG-3' and R, 5'-GTATCGATGCCCACCCTCTAGAGGCCGAGGCGGCCGACA-3' which allow recombination in the linear plasmid pGADT7-Rec when introduced in yeast. Resulting construct carrying Amp^R ^and *LEU *selective characteristics allows expression of a GAL4 Activation Domain (AD)-VvMYB5b fusion protein. The *VvMYC1 *coding sequence (gene accession EU447172) was cloned into the pGBKT7 vector between *Eco*RI and *Pst*I restriction sites [22]. The two-hybrid experiment was conducted using the Clontech BD Matchmaker™ Library Construction & Screening Kit (BD Bioscience) according to the manufacturer's instructions. Yeast strain AH109 was transformed with pGBKT7-*VvMYC1 *construct, *VvMYB5b^R ^*and *VvMyb5b^L ^*PCR products and the effector plasmid pGADT7-Rec, as described in [22]. Transformants were selected on SD- Trp^-^Leu^- ^medium, to make sure that both effector plasmids were indeed integrated to yeast. Transcriptional activation of the reporter gene *LacZ *was evaluated by monitoring β-galactosidase activity.

## Competing interests

The authors declare that they have no competing interests.

## Authors' contributions

IH performed most experiments in this paper and wrote the initial manuscript draft. LD cloned VvMYB5bR and VvMYB5bL sequences and transformed tobacco. JB, AM, FR and CTM participated in experiments (dual luciferase assays, tobacco transformation, gel shift assays and qRT-PCR, respectively). BG and TG contributed to analysis and interpretation of structural data. VL, FB and LD conceived the study, participated in the preparation and finalization of the manuscript. EG has revised the manuscript critically for intellectual content. All authors read and approved the final manuscript.

## Authors' information

IH present address: Groupe de Recherche en Physiologie végétale (GRPV), Earth and Life Institute (ELI), Université catholique de Louvain (UCL), B-1348 Louvain-la-Neuve, Belgium.

## Supplementary Material

Additional file 1 **Detection of the *in vitro *synthesized VvMYB5b^R/L ^proteins**. Both proteins were produced by the *in vitro *transcription and translation method with the TnT T7 quick system for the PCR DNA system (Promega, Charbonnières, France) according to the manufacturer's instruction. The coding sequences were amplified with Turbo-*Pfu *(Stratagene) using the following primers pairs: F, 5'-AGATCCTAATACGACTCACTATAGGGAGCCACCATGAGGAATGCATCCTCAGCA and R, 5'-(T)_32_TCAGAACCGCTTATCAGGTTG. The PCR products were used as template. A 5 μl aliquot of the reagent was used for SDS-PAGE. Separated proteins were transferred onto a nitrocellulose membrane and detected using the Transcend non-radioactive translation detection system (Promega, Charbonnières, France). MW in kDa corresponds to the Page ruler prestained #SM0671 protein ladder (Fermentas). Click here for file

Additional file 2 **Anthocyanin contents in flowers of control and transgenic plants**. Anthocyanin pigments were extracted with 1% HCl in methanol in the dark. The anthocyanin concentration is expressed as the absorbance units at 530 nm per gram of fresh tissue weight. Data are the mean of three replicates, and results from two independent transgenic lines are indicated. ND: not detected. Asterisk indicates values that significantly differ from the control (*P *< 0.05; student's *t *test). Click here for file
