# Supplementary figures and images for: A single amino acid change within the R2 domain of the VvMYB5b transcription factor modulates affinity for protein partners and target promoters selectivity
Source: BMC Plant Biol. 2011 Aug 23;11:117. doi: 10.1186/1471-2229-11-117 (PMC3240579; doi:10.1186/1471-2229-11-117)

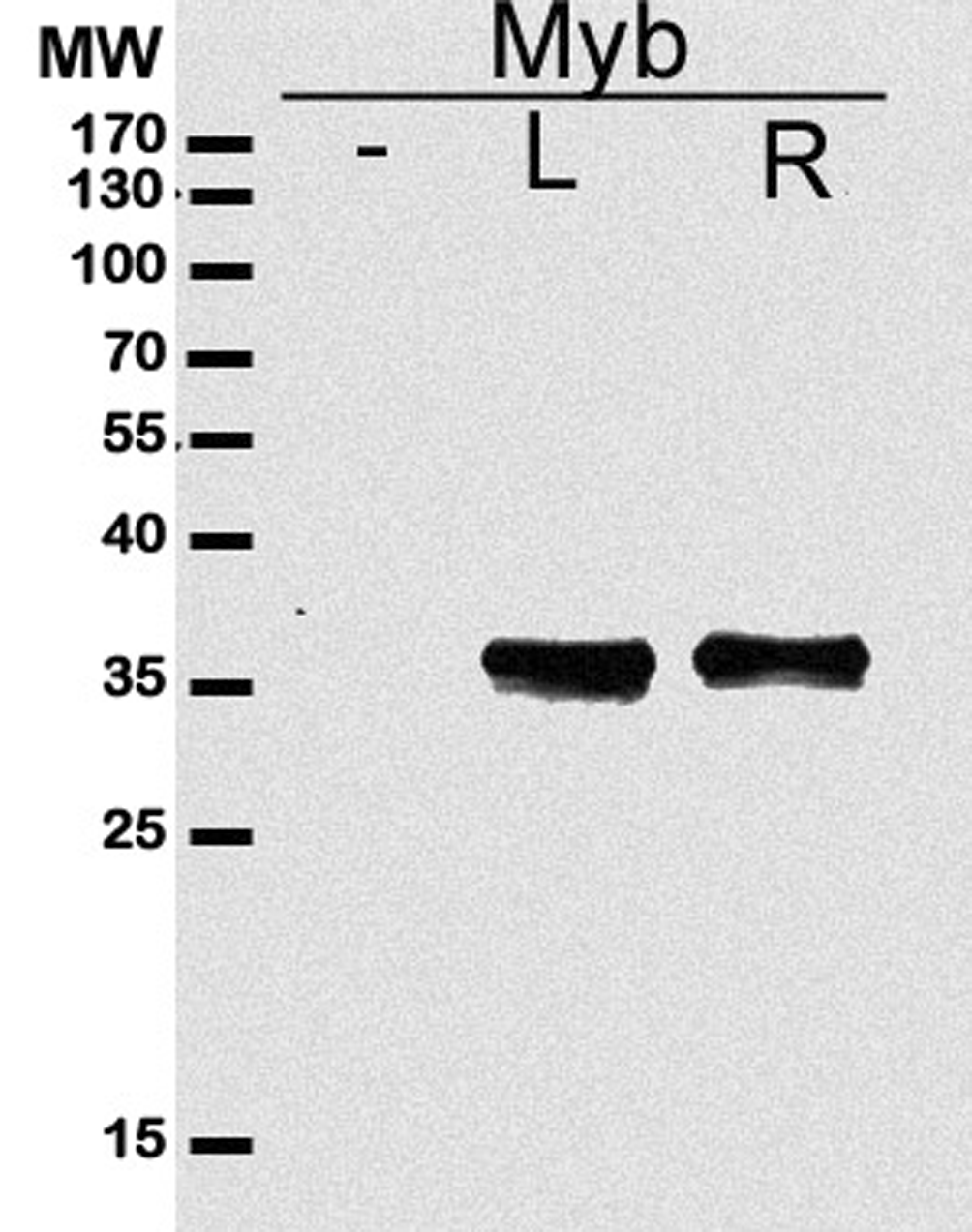

Supplement: Additional file 1 — Detection of the in vitro synthesized VvMYB5bR/L proteins. Both proteins were produced by the in vitro transcription and translation method with the TnT T7 quick system for the PCR DNA system (Promega, Charbonnières, France) according to the manufacturer's instruction. The coding sequences were amplified with Turbo-Pfu (Stratagene) using the following primers pairs: F, 5'-AGATCCTAATACGACTCACTATAGGGAGCCACCATGAGGAATGCATCCTCAGCA and R, 5'-(T)32TCAGAACCGCTTATCAGGTTG. The PCR products were used as template. A 5 μl aliquot of the reagent was used for SDS-PAGE. Separated proteins were transferred onto a nitrocellulose membrane and detected using the Transcend non-radioactive translation detection system (Promega, Charbonnières, France). MW in kDa corresponds to the Page ruler prestained #SM0671 protein ladder (Fermentas). [file 1471-2229-11-117-S1.TIFF]

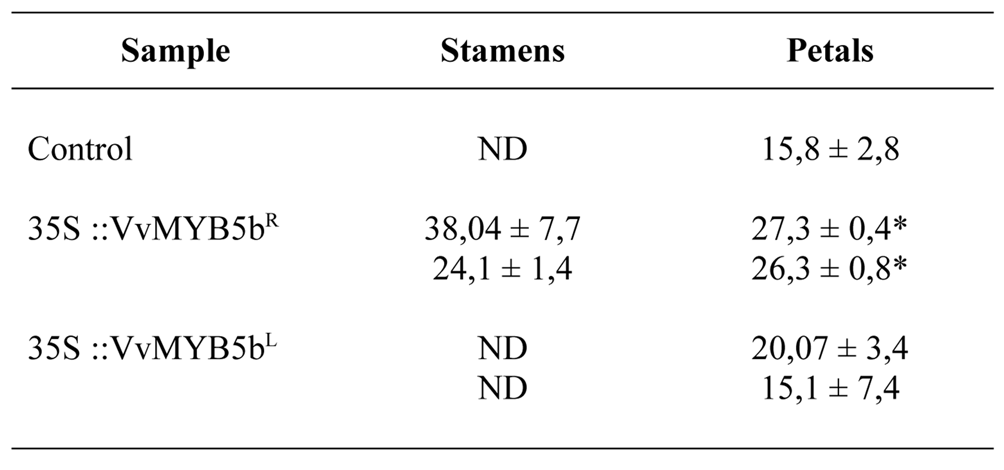

Supplement: Additional file 2 — Anthocyanin contents in flowers of control and transgenic plants. Anthocyanin pigments were extracted with 1% HCl in methanol in the dark. The anthocyanin concentration is expressed as the absorbance units at 530 nm per gram of fresh tissue weight. Data are the mean of three replicates, and results from two independent transgenic lines are indicated. ND: not detected. Asterisk indicates values that significantly differ from the control (P < 0.05; student's t test). [file 1471-2229-11-117-S2.TIFF]
